# A rare case of gastrointestinal diffuse large B cell lymphoma with cold agglutinin disease presentation

**DOI:** 10.1002/kjm2.12953

**Published:** 2025-02-19

**Authors:** Chin‐Mu Hsu, Bi‐Hua Du, Ching‐Fang Hsu, Hui‐Hua Hsiao

**Affiliations:** ^1^ Division of Hematoloty‐Oncology, Department of Internal Medicine Kaohsiung Medical University Hospital Kaohsiung Taiwan; ^2^ Cancer Center Kaohsiung Medical University Hospital Kaohsiung Taiwan; ^3^ Faculty of Medicine Kaohsiung Medical University Kaohsiung Taiwan

Dear Editor,

Cold agglutinin disease (CAD) is an autoimmune hemolytic anemia (AIHA) disorder generally caused by IgM auto‐antibodies to red blood cells which exhibit reactivity at 4°C. It can be happened idiopathically or secondary to other diseases/conditions, such as specific viral infections, infectious mononucleosis, lymphoproliferative disorders.^1^ Diffuse large B cell lymphoma (DLBCL) accounting for the most common subtype of adult non‐Hodgkin lymphoma (NHL), is rarely associated with CAD.^2^, ^3^ Here we report a stage IV gastrointestinal DLBCL with initial presentation of CAD.

This 40 years old female suffered from marked anemia during health exam in October 2020. There is no fever, no lymphadenopathy with some GI upset only. The laboratory test revealed: WBC 5220/μL, Hg 7.8 g/dL, plt 341,000/μL, reticulotycte 3.4%. Coomb's test showed positive result with cold antibody noted. Cold Agglutinin titer showed 256:1 with negative of mycoplasma titer. The CAD was impressed by the test and CT was arranged due to abdomen discomfort and to survey the possibility of lymphoma. CT revealed colon mass with splenomegaly. Biopsy of colon revealed DLBCL. PET‐CT scan showed stage IV disease with lymphoma involving colon, spleen, axillaries, and para‐aortic areas. She received 6 cycles of Rituximab‐EPOCH regimen followed by auto‐stem cell transplant in August 2021 with CR achieved. However, DLBCL relapsed with CNS involvement in March 2023. After multiple salvage therapies, the patient expired in April 2024 due to progression of disease.

The majority of AIHA are medicated by warm‐reactive auto‐antibodies, which displaying reactivity at 37°C by usually IgG antibodies. CAD, accounting around 13%–15% of AIHA, can be primary or secondary to neoplastic growth of B cell clone. Economopoulos et al. reviewed 370 patients of NHL and found only 23 (6.2%) had AIHA and only 4 (1.1%) had CAD.^3^ Berentsen et al. found 76% of patients with chronic CAD had underlying of lymphomas, of which, most of them were lymphoplasmacytic lymphoma, marginal zone lymphoma and small lymphocytic lymphoma/chronic lymphocytic leukemia.^4^ DLBCL with CAD is rarely be reported. The outcomes of DLBCL with CAD is uncertain, with some reports showing aggressive pattern in DLBCL with bone marrow involvement.^5^ However, clinical presentation, optimal treatment strategy and outcomes warranted further information to draw a clear picture. In summary, we report a rare presentation of CAD in DLBCL and highlight the need for lymphoma survey in such kind of patients.

## CONFLICT OF INTEREST STATEMENT

The authors declare no conflict of interest.

## Data Availability

The data that support the findings of this study are available from the corresponding author upon reasonable request.
